# Bone marrow mesenchymal stem cell-derived exosomal microRNA-381-3p alleviates vascular calcification in chronic kidney disease by targeting NFAT5

**DOI:** 10.1038/s41419-022-04703-1

**Published:** 2022-03-28

**Authors:** Yingjie Liu, Yan Guo, Shumin Bao, Hongdong Huang, Wenhu Liu, Weikang Guo

**Affiliations:** 1 Department of Nephrology, Faculty of Kidney Diseases, Beijing Friendship Hospital, Capital Medical University, Beijing, China; 2 Department of Nephrology, Faculty of Kidney Diseases, Shijingshan teaching hospital of Capital Medical University, Beijing Shijingshan Hospital, Beijing, China; 3 Department of Nephrology, Faculty of Kidney Diseases, Beijing Tongren Hospital, Capital Medical University, Beijing, China

**Keywords:** Mesenchymal stem cells, Calcification, End-stage renal disease

## Abstract

Vascular calcification (VC) is a significant complication of chronic kidney disease (CKD) and cellular apoptosis is one of the intricate mechanisms of VC. Bone marrow mesenchymal stem cell-derived exosome (BMSC-Exo) alleviates VC, but the mechanism remains unclear. We investigated the mechanism of BMSC-Exo using high phosphate stimulated Human aortic smooth muscle cells (HA-VSMCs) and 5/6 subtotal nephrectomy (SNx) rat models. We demonstrated that the effect of BMSC-Exo on the inhibition of cellular apoptosis and calcification partially depended on exosomal microRNA-381-3p (miR-381-3p) both in vivo and in vitro, and confirmed that miR-381-3p could inhibit Nuclear Factor of Activated T cells 5 (NFAT5) expression by directly binding to its 3′ untranslated region. Additionally, we found that severe calcification of arteries in dialysis patients was associated with decreased miR-381-3p and increased NFAT5 expression levels. Collectively, our findings proved that BMSC-Exo plays anti-calcification and anti-apoptosis roles in CKD by delivering enclosed miR-381-3p, which directly targets NFAT5 mRNA, and leads to a better understanding of the mechanism of CKD-VC.

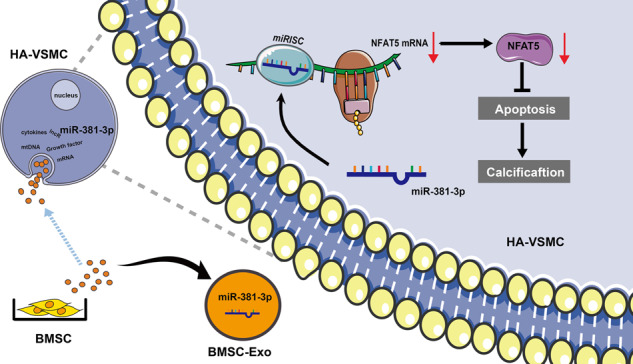

## Introduction

Cardiovascular events are the leading cause of death in patients with chronic kidney disease (CKD) [1, 2], and their incidence is much higher than that of age-matched non-CKD populations [3]. Vascular calcification (VC) is popular in CKD patients and is closely related to cardiovascular events such as myocardial infarction and sudden cardiac death [4, 5]. Therefore, prevention and treatment of VC plays a key role in reducing the occurrence of cardiovascular events and improving the prognosis of CKD patients. However, there is currently a lack of treatments to alleviate CKD-VC in clinical practice.

Among the pathogenic factors of vascular calcification, hyperphosphatemia is considered to be the most important one [6, 7]. Studies have shown that vascular smooth muscle cell (VMSC) apoptosis caused by hyperphosphatemia plays a crucial role in CKD-VC [8–10]. Apoptotic bodies result in the deposition of calcium and phosphate, which develop into the core of calcification and play a crucial role in the development of VC [11–13]. Therefore, understanding the mechanisms contributing to VSMC apoptosis is essential to prevent and decelerate the progression of CKD-VC.

Recently, the therapeutic effects of mesenchymal stem cells (MSCs) have obtained great more focus, and exosomes secreted by MSCs are of particular interest [14]. Exosomes are small vesicles (40–150 nm in diameter) released by various cells after the fusion of the multivesicular bodies with the cell membrane [15, 16]. Considering the significant substances enclosed in exosomes including proteins, RNAs (miRNA, lncRNA, and mRNA), and lipids, exosomes were proved to function in various processes that include tissue damage repair [17–19], regulation of apoptosis [20–22], suppression of inflammatory responses [23], and modulation of autophagy [24]. As previously reported, BMSC-Exo exerted a protective role in myocardial ischemia/ reperfusion [25], hypoxia induced myocardial injury [26], wound-healing process in diabetic foot ulcer [27], intervertebral disc degeneration [28] as well as liver failure [29]. We also found that BMSC-Exo can significantly reduce the calcification of VMSC caused by hyperphosphatemia in previous studies [30], indicating that BMSC-Exo may be valuable in the treatment of CKD-VC, however the possible underlying mechanisms remains unclear.

In this study, we aim to investigate the anti-apoptosis and anti-calcification effects of BMSC-Exo in CKD-VC and clarify its mechanism, which not only leads to a better understanding of the protective role of BMSC-Exo, but also provides a potential therapeutic strategy for CKD-VC.

## Results

### BMSC-Exo inhibited high Pi-induced apoptosis and calcification in HA-VSMCs

**Fig. 1:**
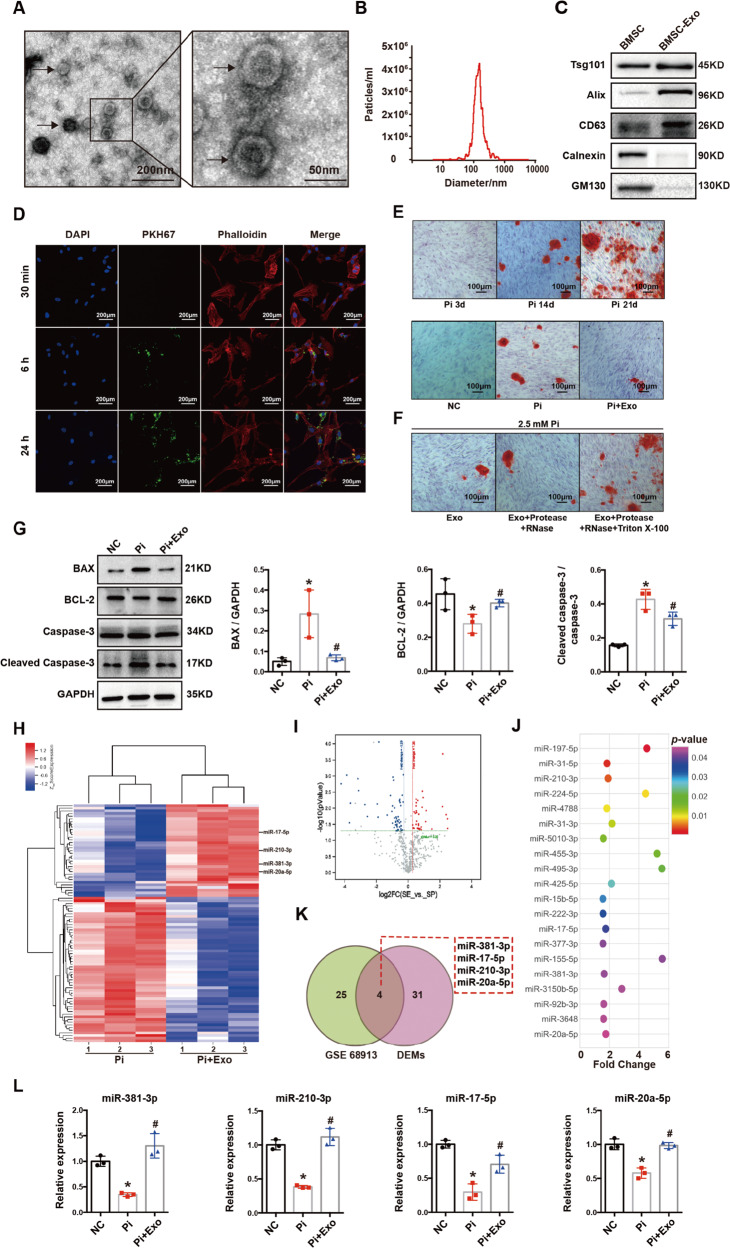
BMSC-Exo inhibited apoptosis and VC in HA-VSMCs. **A** The ultrastructure of exosomes (Black Arrow) was observed by TEM (left panel scale bar = 200 nm, right panel scale bar = 50 nm). **B** The particle size was analyzed by NTA. **C** The expression levels of Tsg 101, Alix, CD63, Calnexin, and GM130 were detected by western blot. **D** The uptake of BMSC-Exo (green fluorescence) by HA-VSMCs was observed by confocal microscope. **E**, **F** Alizarin Red S staining was used to evaluate the calcium deposits of HA-VSMCs under different treatments (scale bar = 100 μm). **G** Western blot was used to detect the protein expression levels of BAX, BCL-2, and cleaved caspase-3 after treatment of BMSC-Exo. **H**, **I** DEMs between Pi and Pi + Exo group were presented by heatmap and volcano plot. **J** Top 20 upregulated miRNAs of DEMs were displayed in Bubble Chart. **K** The intersection of GSE 68913 and the DEMs was presented by Venn graph. **L** Expression of different miRNAs in NC, Pi, and Pi + Exo groups was detected by RT-qPCR. *P < 0.05 compared with the NC group; ^#^*P* < 0.05 compared with the Pi group.

Firstly, we validated the quality of BMSC-Exo using several methods. The isolated fractions were clearly displayed as a typical “cup-like” structure under transmission electron microscopy (TEM) (Fig. 1A). Nanoparticle tracking analysis (NTA) indicated that BMSC-Exo was nanoparticles with a diameter around 139.6 nm, and the main peak accounted for 94.3% of the particles (Fig. 1B). Exosome markers (CD63, Tsg 101, and Alix) were detected in the isolated fractions by western blot. Conversely, cytoplasmic biomarkers (Calnexin and GM130) were absent in the exosomes (Fig. 1C). Exosome uptake assay presented that PKH67 labeled (green fluorescence) BMSC-Exo was visualized around the nuclei of HA-VSMCs (Fig. 1D), which confirms the internalization of BMSC-Exo by HA-VSMCs.

We stimulated HA-VSMCs with 2.5 mM Pi for 3, 14, or 21 days and found that obvious calcification appeared after 14 days’ stimulation (Fig. 1E and Supplementary Fig. S1A). We then analyzed the effect of BMSC-Exo on the calcification of HA-VSMCs. Treatment of 2.5 mM Pi for 14 days significantly increased calcium deposits compared with the NC group, whereas fewer calcium deposits were observed in Pi + Exo group (Fig. 1E and Supplementary Fig. S1B), which indicated that BMSC-Exo inhibited calcification induced by high Pi. Furthermore, the results (Fig. 1F and Supplementary Fig. S1C) demonstrated that RNase and protease treatment alone did not affect the inhibitory role of BMSC-Exo in calcification, but it was significantly abolished by combined use of Triton X-100 (Fig. 1F), which indicated that exosome-enclosed substances rather than RNAs/proteins from other sources were responsible for the calcification-protective effects of BMSC-Exo.

In addition, we detected the expression levels of apoptotic indicators in HA-VSMCs. Western blot analysis showed a marked increase in BAX and cleaved caspase-3 and a significant decrease in BCL-2 after treatment with 2.5 mM Pi. After administration with BMSC-Exo, BAX and cleaved caspase-3 expression was reduced, while BCL-2 expression increased (Fig. 1G). These results suggest that BMSC-derived exosomes ameliorated Pi-induced apoptosis and VC in HA-VSMCs.

### The inhibitory role of BMSC-Exo on the calcification partially depended on miR-381-3p

**Fig. 2:**
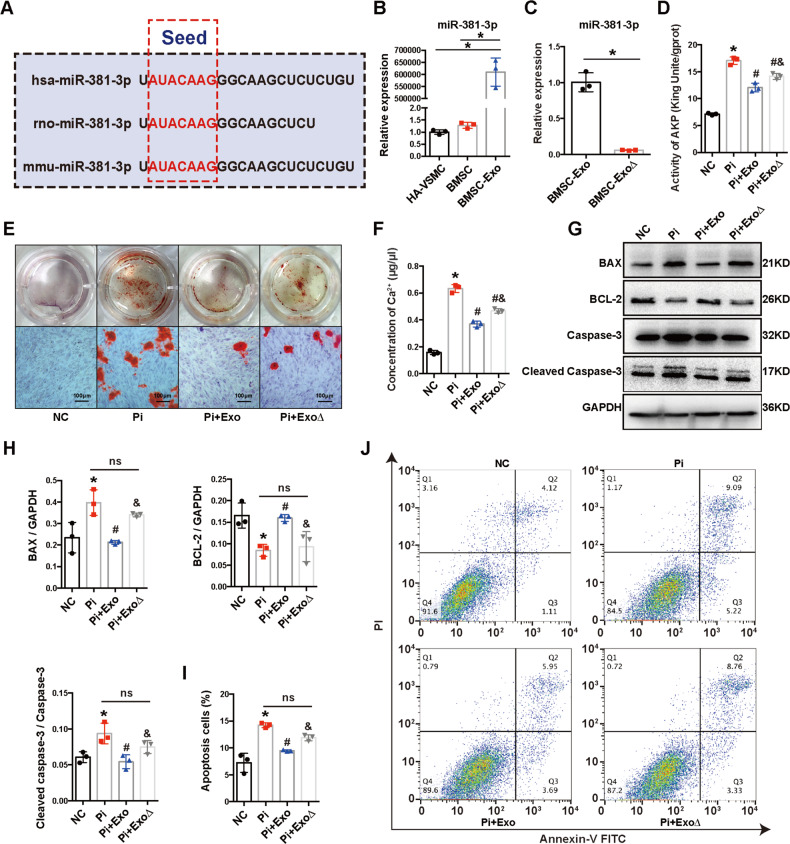
The effect of BMSC-Exo on the inhibition of calcification depended on miR-381-3p. **A** Sequences and seed region of miR-381-3p in different species. **B**, **C** The expression of miR-381-3p was detected by RT-qPCR, *P < 0.05. **D**–**F** AKP activity, Alizarin Red S Stain (scale bar = 100 μm) and Ca^2+^ content assay were used under treatment of different exosomes, *P < 0.05 compared with the NC group; ^#^*P* < 0.05 compared with the Pi group; ^&^*P* < 0.05 compared with the Pi + Exo group. **G**, **H** Protein levels of BAX, BCL-2 and cleaved caspase-3 were detected under treatment of different exosomes, *P < 0.05 compared with the NC group; ^#^*P* < 0.05 compared with the Pi group; ^&^*P* < 0.05 compared with the Pi + Exo group. **I**, **J** Apoptosis was measured by flow cytometry, *P < 0.05 compared with the NC group; ^#^*P* < 0.05 compared with the Pi group, ^&^*P* < 0.05 compared with the Pi + Exo group.

MiRNA microarray analysis was performed to identify differentially expressed miRNAs (DEMs) between the Pi and Pi + Exo groups in HA-VSMCs (FC > 1.2, *P* < 0.05, Fig. 1H, I); the top 20 upregulated miRNAs of DEMs were presented in Fig. 1J. Then we selected GSE 68913 (non-coding RNA profiling array for VSMCs between CKD rats and control) published in Gene Expression Omnibus (GEO) as an additional dataset to narrow down the possible candidates. Four upregulated miRNAs were observed to be shared in the two selected data sets (Fig. 1K), including miR-381-3p, miR-17-5p, miR-210-3p and miR-20a-5p. Then RT-qPCR was used to detect the expression levels of the four miRNAs among NC, Pi and Pi + Exo groups (Fig. 1L). Among the four miRNAs, miR-381-3p decreased significantly after stimulation with 2.5 mM Pi and increased most noticeably after treatment with BMSC-Exo. Therefore, miR-381-3p was selected as a target for further study. Moreover, sequences of miR-381-3p were highly conserved with the same seed sequence among different species as presented in Fig. 2A.

We evaluated the expression of miR-381-3p in HA-VSMCs, BMSCs, and BMSC-Exo, and found that miR-381-3p was enriched in BMSC-Exo (Fig. 2B). We then transfected BMSCs with miR-381-3p inhibitors (200 nM) to silence its expression in exosomes (BMSC-Exo^∆^). Our data confirmed that the expression level of miR-381-3p in BMSC-Exo^∆^ was significantly decreased compared with that in BMSC-Exo (Fig. 2C). Next, we analyzed the biological function of BMSC-Exo^∆^ on calcification. Alizarin Red S staining, Ca^2+^ content assay, and AKP activity analysis indicated that calcium deposits were significantly reduced after the treatment with BMSC-Exo and BMSC-Exo^∆^, but the inhibition of BMSC-Exo^∆^ on calcification was significantly poor compared with that of BMSC-Exo (Fig. 2D–F). Next, we evaluated the effect of miR-381-3p on cellular apoptosis in HA-VSMCs. Fig. 2G, H indicated that the inhibitory effects on BAX and cleaved caspase-3, and the enhancing effects on BCL-2 expression had been abolished after knocking down miR-381-3p expression in BMSC-Exo. Flow cytometry analysis confirmed that BMSC-Exo significantly inhibited apoptosis, but BMSC-Exo^∆^ had no such effect (Fig. 2I, J). These results demonstrated that miR-381-3p was responsible for the anti-calcification and anti-apoptosis effects of BMSC-Exo.

### MiR-381-3p inhibited calcification and apoptosis in HA-VSMCs by directly targeting NFAT5

**Fig. 3:**
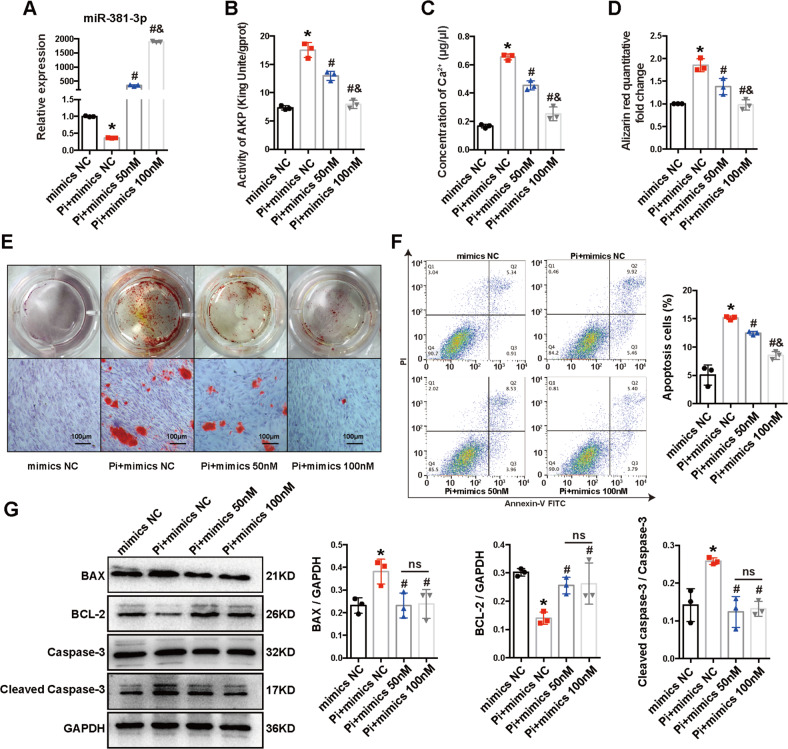
Effects of miR-381-3p on VC and apoptosis in HA-VSMCs. **A** The expression of miR-381-3p in HA-VSMCs was detected by RT-qPCR. **B**, **C** AKP activity and Ca^2+^ content in HA-VSMCs were detected after transfected with miR-381-3p mimics. **D**, **E** Mineral deposits in HA-VSMCs were stained by Alizarin Red S after transfected with miR-381-3p mimics (scale bar = 100 μm). **F** Apoptosis was measured by flow cytometry. **G** The protein expression of BAX, BCL-2 and cleaved caspase-3 was detected by western blot. *P < 0.05 compared with the mimics NC group; ^#^*P* < 0.05 compared with the Pi + mimics NC group.

To further analyze the effect of miR-381-3p on calcification, we used miR-381-3p mimics (50 nM and 100 nM) to overexpress miR-381-3p in HA-VSMCs. Results in Fig. 3A demonstrated that the expression level of miR-381-3p was successfully upregulated in a concentration dependent method. Next, we observed the calcification of HA-VSMCs transfected with mimics. The results of AKP activity, Ca^2+^ content, and Alizarin Red S Staining assays indicated that calcium deposits were significantly decreased in a dose dependent manner after transfected with miR-381-3p mimics compared with the Pi group (Fig. 3B–E). We then investigated whether miR-381-3p could affect the apoptosis of HA-VSMC. Flow cytometry analysis revealed that the inhibitory effect on apoptosis became more pronounced with increasing concentration of miR-381-3p mimics (Fig. 3F). Next, we detected the protein biomarkers of apoptosis by the transfection of mimics. The protein expression level of BAX and cleaved caspase-3 were significantly decreased by mimics, while the expression of BCL-2 was increased compared with the Pi group, but no significant difference was observed between the two subgroups with different concentration of mimics (Fig. 3G). Taken together, these results indicated that miR-381-3p significantly inhibited calcification and apoptosis of HA-VSMCs.

**Fig. 4:**
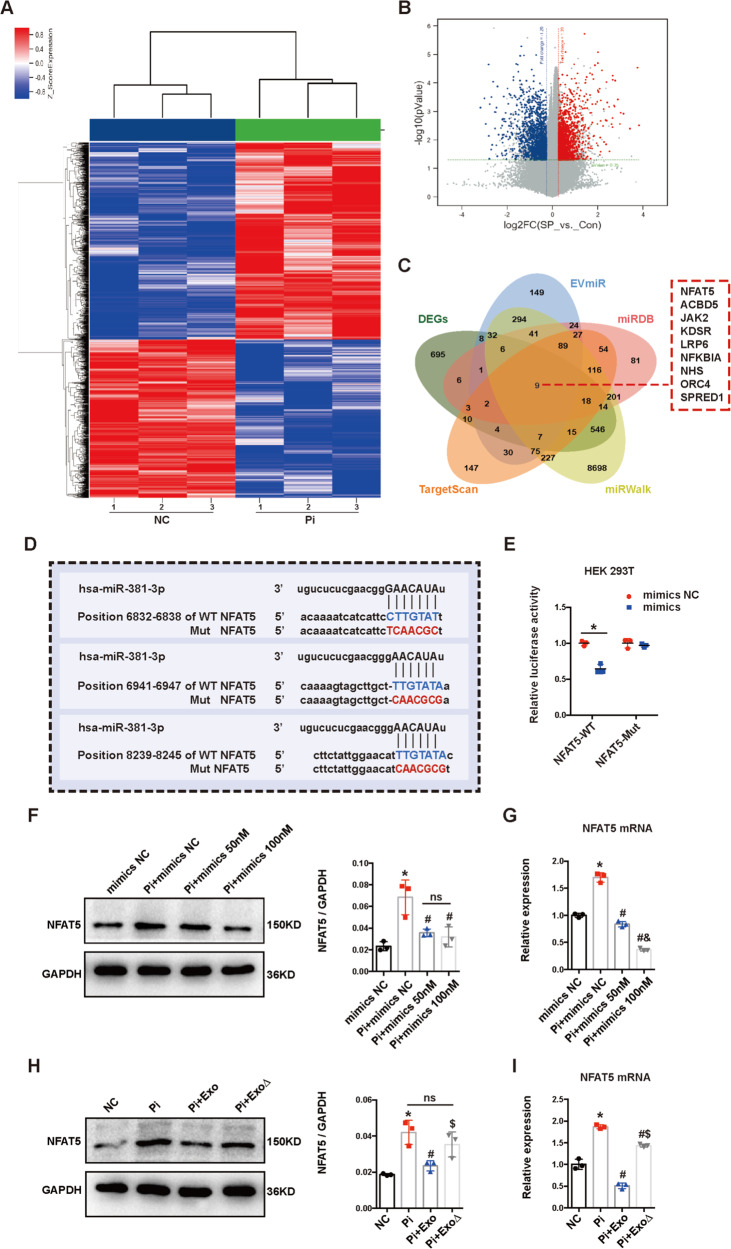
MiR-381-3p inhibited NFAT5 by directly targeting its 3’UTR. **A**, **B** DEGs between NC and Pi group were presented by heatmap and volcano plot. **C** The intersection of different online databases and DEGs was presented by Venn graph. **D** The binding sites of miR-381-3p and NFAT5 were predicted by TargetScan. **E** The combination of miR-381-3p and NFAT5 was verified by dual-luciferase reporter gene assay, *P < 0.05. **F**, **G** The protein and mRNA expression levels of NFAT5 in HA-VSMCs were detected after transfection of miR-381-3p mimics, *P < 0.05 compared with the mimics NC group; ^#^*P* < 0.05 compared with the Pi + mimics NC group; ^&^*P* < 0.05 compared with the Pi + mimics 50 nM group. **H**, **I** The protein and mRNA expression levels of NFAT5 in HA-VSMCs were detected under treatment of different exosomes, *P < 0.05 compared with the NC group; ^#^*P* < 0.05 compared with the Pi group; ^$^*P* < 0.05 compared with the Pi + Exo group.

To further determine the possible targets of miR-381-3p, we performed a microarray assay between NC and Pi group (FC ≥ 1.2, *P* < 0.05, Fig. 4A, B). We used several online target gene prediction databases (including TargetScan, miRDB, EVmiR, and miRWalk) to narrow down the possible candidates. Nine target genes were observed as the intersection of four online databases and DEGs, including NFAT5, ACBD5, JAK2, KDSR, LRP6, NFKBIA, NHS, ORC4, and SPRED1 (Fig. 4C). Previous reports showed that NFAT5 (Nuclear Factor of Activated T cells 5) may play a crucial role in VC. Therefore, we assumed NFAT5 may be a target gene in miR-381-3p-mediated regulation of calcification. The binding sites of NFAT5 and miR-381-3p predicted by TargetScan were presented in Fig. 4D. Dual-luciferase reporter assay was performed to further validate the hypothesis. The wildtype (Wt) and mutant (Mut) NFAT5 3′ untranslated region (UTR) sequences were synthesized (Fig. 4D). Luciferase activity was significantly attenuated by miR-381-3p mimics with Wt 3′UTR sequences, which was not observed in mimics with the Mut sequences (Fig. 4E), indicating that miR-381-3p could combine with 3′UTR sequence of the NFAT5 mRNA directly. Next, we evaluated the changes in protein and mRNA expression levels of NFAT5 in HA-VSMCs upon transfection with mimics. Both protein and mRNA levels of NFAT5 were downregulated by mimics, indicating that miR-381-3p regulated NFAT5 expression at the mRNA and protein levels (Fig. 4F, G). Considering that miR-381-3p is enriched in BMSC-Exo, we also proved that NFAT5 mRNA and proteins expression levels were significantly downregulated upon application of BMSC-Exo in HA-VSMCs. Furthermore, the expression of NFAT5 was also decreased at the mRNA level in HA-VSMCs stimulated with BMSC-Exo^∆^ compared with Pi group, which was still significantly higher than that in the BMSC-Exo group. However, the protein expression of NFAT5 stimulated by BMSC-Exo^∆^ indicated no statistical difference compared with the Pi group (Fig. 4H, I). These results demonstrated that miR-381-3p regulated NFAT5 expression by directly targeting its mRNA 3′UTR sequence.

SiRNA targeting human NFAT5 (Si-NFAT5) was transfected into HA-VSMCs to further clarify whether knocking down NFAT5 attenuated apoptosis and calcification of HA-VSMCs. Firstly, the mRNA and protein expression of NFAT5 were significantly decreased by transfection of si-NFAT5 (Supplementary Fig. S1D, E). However, the miR-381-3p expression was unaffected (Supplementary Fig. S1F). The results of Alizarin Red S staining, Ca^2+^ content, and AKP activity assays indicated that the calcium deposits induced by Pi were significantly reduced by knocking down NFAT5 (Supplementary Fig. S1G, H). Moreover, flow cytometry suggested that apoptosis induced by high Pi was significantly reduced by knocking down NFAT5 (Supplementary Fig. S1I). Additionally, the mRNA and protein expression levels of apoptotic indicators BAX and cleaved caspase-3 were significantly decreased, while BCL-2 was increased by Si-NFAT5 (Fig. S1J-L). These results indicated that knock-down of NFAT5 inhibited apoptosis and calcification induced by high Pi.

**Fig. 5:**
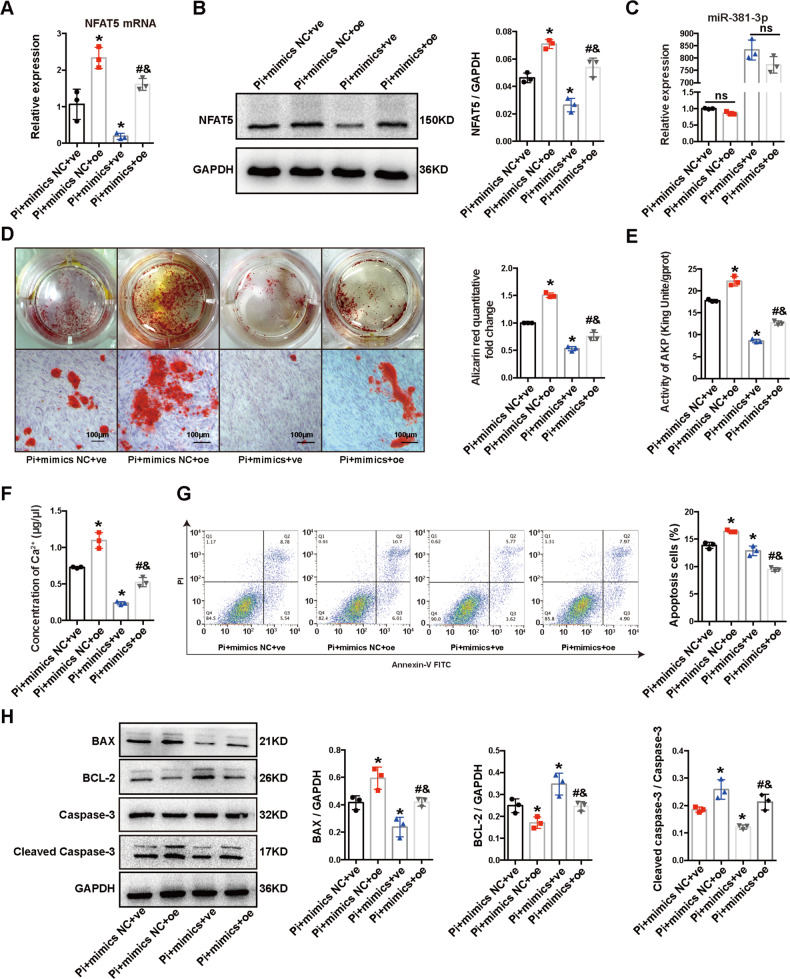
MiR-381-3p inhibited apoptosis and VC by targeting NFAT5. **A**, **B** The mRNA and protein expression levels of NFAT5 were detected after transfected with oe-NFAT5 or (and) miR-381-3p mimics. **C** Expression of miR-381-3p was detected by RT-qPCR. **D**–**F** Alizarin Red S staining, Ca^2+^ content, and AKP activity were detected after transfected with oe-NFAT5 or (and) miR-381-3p mimics (scale bar = 100 μm). **G** Apoptosis was measured by flow cytometry. **H** The protein expression of BAX, BCL-2, and cleaved caspase-3 was detected after transfected with oe-NFAT5 or (and) miR-381-3p mimics. *P < 0.05 compared with the Pi + mimics NC + ve group; ^#^*P* < 0.05 compared with the Pi + mimics NC + oe group; ^&^*P* < 0.05 compared with the Pi + mimics + ve group.

Then we assessed whether the inhibitory role of exo-miR-381-3p depend on its regulation on NFAT5. MiR-381-3p mimics (100 nM) and human NFAT5 over-expression plasmid (oe-NFAT5) were co-transfected into HA-VSMCs. Expression of NFAT5 was significantly increased at mRNA and protein levels after transfected with oe-NFAT5 (Fig. 5A, B), but expression of miR-381-3p remained unchanged (Fig. 5C). MiR-381-3p mimics reversed the over-expression of NFAT5 caused by oe-NFAT5 at the mRNA and protein levels (Fig. 5B). Apoptosis and calcification were also assessed after co-transfection. As shown in Fig. 5D–G, Alizarin Red S staining, Ca^2+^ content assay, AKP activity assay and flow cytometry analysis revealed that calcium deposits and apoptosis ratio in HA-VSMCs were significantly increased by oe-NFAT5, which were partially reversed by miR-381-3p mimics. The decreased calcium deposits and apoptosis ratio caused by mimics could also be partially reversed by oe-NFAT5. Furthermore, we detected apoptotic indicators after co-transfection, the results indicated that the decreased expression of BAX, cleaved caspase-3 and increased of BCL-2 caused by miR-381-3p mimics were significantly reversed by oe-NFAT5 (Fig. 5H). Collectively, the cellular apoptosis and calcification were significantly aggravated by oe-NFAT5, and the inhibitory effects of miR-381-3p mimics were largely attenuated by oe-NFAT5, which indicated NFAT5 was crucial for the anti-calcification and anti-apoptosis effects of exosomal miR-381-3p.

### The therapeutic effects of BMSC-Exo on apoptosis and VC in SNx rats

**Fig. 6:**
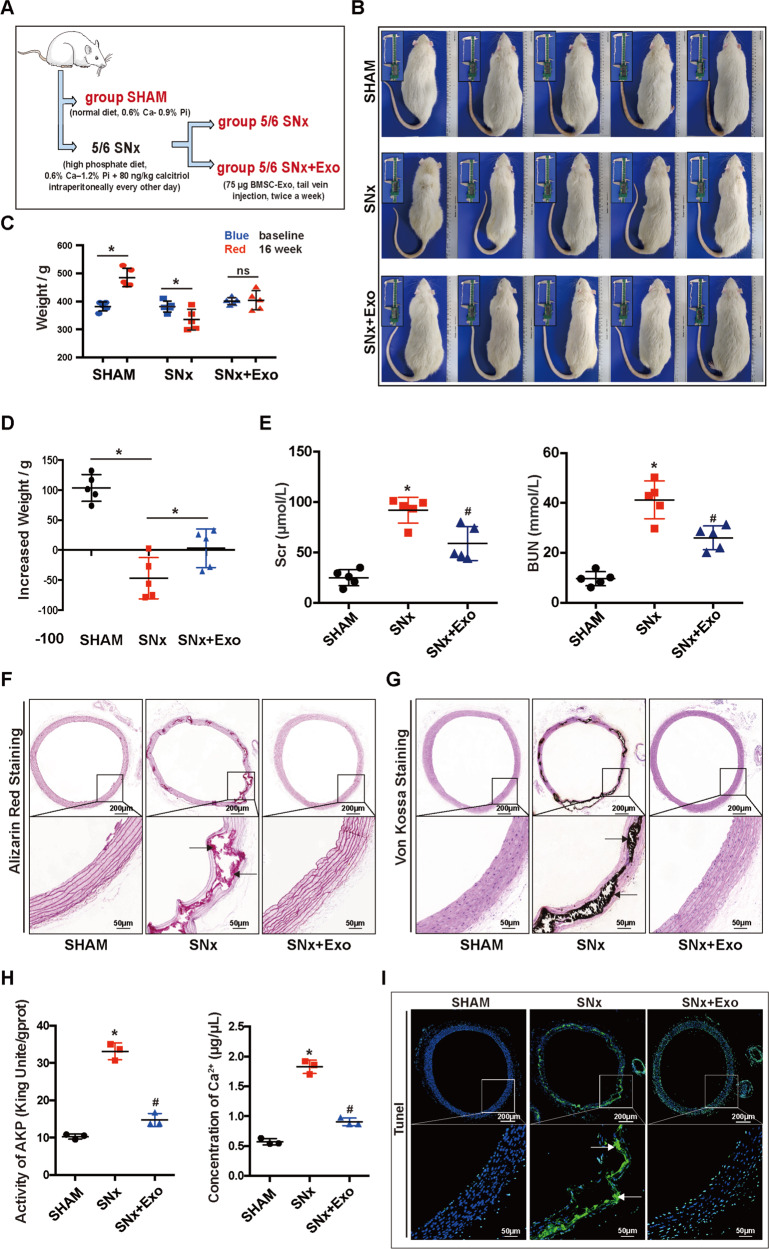
The therapeutic effects of BMSC-Exo on rat survival, renal function, cellular apoptosis and VC in 5/6 SNx rats. **A** The pattern diagram of the research design in vivo. **B** Photograph of Sprague Dawley rats in the SHAM and SNx groups after treatment of BMSC-Exo. **C**, **D** Weight changes of rat in SHAM, SNx and SNx + Exo group were presented by histogram, *P < 0.05. **E** Concentrations of Scr and BUN in rats were presented by histogram. **F**, **G** Thoracic aorta of rats were stained by Alizarin Red S and V-K (upper panel scale bar = 200 μm, lower panel scale bar = 50 μm). (H) Ca^2+^ content and AKP activity were detected in SHAM, SNx, and SNx + Exo groups. **I** TUNEL staining was used to detect apoptosis in the thoracic aorta of rats (upper panel scale bar = 200 μm, lower panel scale bar = 50 μm). Positive staining, indicated with arrows, denotes the region of calcification or apoptosis in the thoracic aorta. *P < 0.05 compared with the SHAM group; ^#^*P* < 0.05 compared with the SNx group.

In this study, a stable VC model was established using 5/6 SNx rats (Fig. 6A). Rats didn’t survive for more than 16 weeks after 5/6 SNx because of the high mortality. The mortality of rats was approximately 37.5% (3/8) in 16 weeks after SNx, which was decreased to 25% (2/8) after tail vein injection of BMSC-Exo. Rats in the SNx group were obviously thinner than those in SNx + Exo and SHAM groups after 16-weeks post-operation (Fig. 6B). Moreover, the body weight of the rats in the SHAM group was increased by 103.6 ± 9.976 g (*N* = 5) compared with their baseline and that of rats in SNx group was decreased by 46.78 ± 15.34 g (*N* = 5). However, changes of bodyweight in the SNx + Exo group were not significant throughout 16-weeks of observation (3.040 ± 14.41 g, *N* = 5) (Fig. 6C, D). Moreover, the levels of Scr and BUN were significantly increased in SNx group compared with SHAM group, and depressed after the administration of BMSC-Exo (Fig. 6E). Therefore, BMSCs-Exo prolonged survival and improved the renal function of SNx rats.

**Fig. 7:**
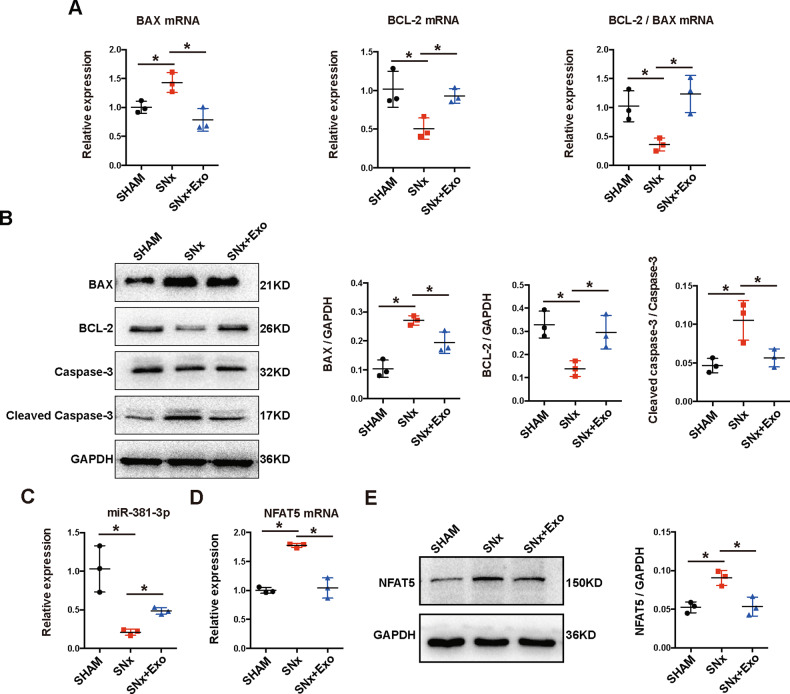
BMSC-Exo inhibited apoptosis of thoracic aorta in 5/6 SNx rats through regulating miR-381-3p/NFAT5 axis. **A**, **B** The mRNA and protein expression levels of BAX, BCL-2, and cleaved caspase-3 were detected in rats. **C** Expression of miR-381-3p was detected by RT-qPCR. **D**, **E** The protein and mRNA expression levels of NFAT5 were detected in rats. *P < 0.05.

Next we investigated the effects of BMSC-Exo on the apoptosis and VC of thoracic aorta in vivo. First, Alizarin Red S and Von Kossa (V-K) staining of thoracic aorta yielded positive results after 16 weeks in all rats with SNx, whereas few calcium deposits were observed in the SHAM group (Fig. 6F, G). Administration of exosomes alleviated the calcification of the thoracic aorta, but thickening of the vascular media was still observed (Fig. 6F, G). To better confirm these findings, we quantified the Ca^2+^ content and AKP activity. The results indicated a significant increase of Ca^2+^ content and AKP activity in the thoracic aorta after SNx, which were alleviated by BMSC-Exo treatment (Fig. 6H). Next, we used TUNEL assay to detect apoptosis in the thoracic aorta. Few apoptotic cells were observed in the SHAM group, whereas a large number of apoptotic cells were distributed along with the vascular media in the SNx group, which was similar to the distribution of calcium deposits. Compared with the SNx group, TUNEL-positive cells were significantly reduced after treatment of BMSC-Exo (Fig. 6I). The results of RT-qPCR and western blot indicated that BMSC-Exo decreased the expression of BAX and cleaved caspase-3, and increased BCL-2 expression at the mRNA and protein levels caused by SNx (Fig. 7A, B), which suggested that BMSC-Exo alleviated SNx-induced apoptosis in the thoracic aorta.

### Exo-miR-381-3p/ NFAT5 axis was involved in anti-calcification effect in vivo

We first evaluated the expression of miR-381-3p and NFAT5 in SNx rats treated with BMSC-Exo. The expression level of miR-381-3p was downregulated while both mRNA and protein levels of NFAT5 were upregulated by SNx, which were reversed by treatment with BMSC-Exo (Fig. 7C–E).

**Fig. 8:**
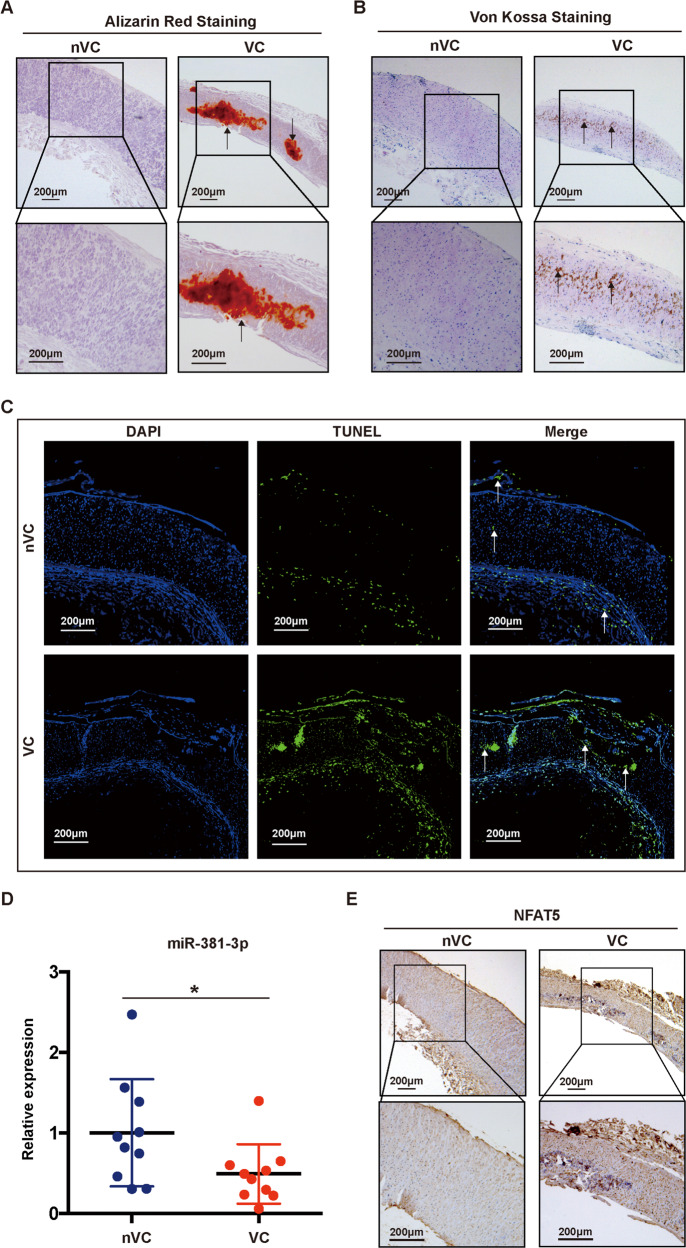
Decreased miR-381-3p expression was associated with severe apoptosis and VC in dialysis patients. **A**, **B** Alizarin Red S staining and V-K staining of arteries obtained from dialysis patients (scale bar = 200 μm). **C** TUNEL staining was used to detect apoptosis of arteries in dialysis patients (scale bar = 200 μm). **D** RT-qPCR was used to detect the expression of miR-381-3p in arteries obtained from dialysis patients, *P < 0.05. **E** The NFAT5 expression of arteries in dialysis patients was detected by immunohistochemistry (scale bar = 200 μm). Positive staining, indicated with arrows, denotes the region of calcification or apoptosis in the arteries.

We then aim to verify the crucial role of exo-miR-381-3p/ NFAT5 axis in dialysis patients. We performed Alizarin Red S and V-K staining of upper extremity arteries preserved in formaldehyde from dialysis patients, which were divided into two groups: the VC group (*n* = 10) and non-VC group (nVC group, *n* = 10) according to the presence of calcium deposits (Fig. 8A, B). As we expected, TUNEL assay revealed that a large number of apoptotic cells were distributed along with the vascular media in the VC group, which was consistent with the result in 5/6 SNx rats (Fig. 8C). RT-qPCR results proved that the expression of miR-381-3p in the vascular tissue of the nVC group was significantly higher than that in the VC group (Fig. 8D), suggesting the possible protective effect of miR-381-3p on VC in dialysis patients. Immunohistochemistry indicated that positive staining of NFAT5 was markedly increased in the VC group, suggesting NFAT5 played a potential deteriorative role in VC (Fig. 8E).

## Discussion

VC is an important pathological basis for cardiovascular disease, which is common in dialysis patients. But how to inhibit VC has become a thorny issue. Our previous study [30] found that BMSC-Exo can inhibit Pi-caused cell calcification in VSMCs. In this study, we further verified that BMSC-Exo ameliorated VC in CKD rats. At the same time, we found that BMSC-Exo delivered miR-381-3p and inhibited its target gene NFAT5, and then inhibited VSMCs apoptosis, thereby exerting its function of inhibiting vascular calcification.

Exosomes represent a potential alternative therapy for many diseases [14]. Compared with cells or cytokines, exosomes are more suitable for the treatment of complex diseases because its stable characteristics with a low risk of severe immune response. Besides, exosomes can be packaged, modified and loaded with more substances in the regulation of biological activities [14]. Until now, the optimal method for the isolation of exosomes is still under debate. Various strategies and techniques (e.g., Polyethylene glycol coprecipitation, density gradient separation, immunoaffinity capture as well as ultracentrifugation) have been used in the isolation of exosomes, but none of them is satisfying for both purity and quantity. Ultracentrifugation is a commonly used method to obtain exosomes, which has been applied in many articles including ours. To be noticed, the application dose of BMSC-Exo in our study depended on the isolation strategy (ultracentrifugation), which should be adjusted appropriately according to the purity and quantity of the exosomes in clinical application. Several studies have demonstrated the vital role of exosomes in the regulation of VC. F Xu *et al*. proved that both VSMCs and melatonin-treated VSMCs derived exosomes could decelerate the osteogenic differentiation of VSMCs via delivering miRNAs [19]. Exosomes isolated from BMSCs could promote functional recovery, regulate apoptosis, and act as a possible target for treating cerebrovascular disease [22, 31]. The preliminary results [30] of our team have demonstrated that BMSC-Exo could ameliorate VC, prevent adipogenic differentiation, and multiple miRNAs were involved in such a biological process.

Numerous microRNAs have been found to participate in the regulation of biological functions, and many of them could be delivered to recipient cells via exosomes. Our data revealed that the application of BMSC-Exo or over-expression of miR-381-3p reduced mortality rates and weight loss of SNx rats, decreased the apoptosis of VSMCs, and inhibited progression of VC. It has been reported that miR-381-3p inhibited cell apoptosis and necroptosis in renal cancer, which is consistent with our observations [32].

In this study, we proposed nine consensus possible target genes of miR-381-3p and validated NFAT5 which has not been extensively studied in the context of VC in molecular biological assays. The human NFAT5 gene was found to be located on chromosome 16q22.1 [33] and could be detected in various tissues including the heart, lung, kidney, brain, and thymus [34, 35]. NFAT5 belongs to the Rel transcription factor family, and is a key factor in the maintenance of cell homeostasis to resist hypertonic environments [36]. Evidence demonstrated that NFAT5 may play a role in apoptosis and VC but the mechanism is still unclear [37, 38].

Previous studies have shown that NFAT5 were regulated by various miRNAs to maintain cell homeostasis and regulate biological functions. MiR-466(a/e)-3p was found to regulate urine concentration in mice by targeting NFAT5 [39]. Additionally, miR-361-3p bound with NFAT5 during transdifferentiation, resulting in activation of several pathways such as JNK, NF-κB and P38 [39, 40]. Our research confirmed that exosomal miR-381-3p regulated the expression of NFAT5 by directly targeting its 3′UTR, which has not been reported before, performing an inhibitory effect on apoptosis and the occurrence of VC in CKD.

In summary, we proposed a new mechanism that BMSC-derived exosomal miR-381- 3p could down-regulate NFAT5, therefore alleviate the cellular apoptosis and VC. This study not only reveals the significant role of miR-381-3p/NFAT5 in regulating apoptosis/VC but also expands our understanding of the regulatory effects of BMSC-Exo on diseases with complex mechanisms such as CKD-VC.

## Materials and methods

### Cell culture and induction of calcification

HA-VSMCs were obtained from ScienCell Research Laboratories (CA, USA), and were cultured in SMC medium containing 2% fetal bovine serum (FBS). HA-VSMCs were stimulated with inorganic phosphate (final concentration 2.5 mM, pH 7.4) (Sigma-Aldrich, MI, USA) to induce calcification [41]. Cells in our experiments were all tested for short tandem repeat analysis and were free of mycoplasma contamination.

### Isolation of exosomes

BMSCs were obtained from ScienCell Research Laboratories (CA, USA), and were cultured in MSC medium containing 10% exosome-depleted FBS (Thermo Fisher, cat. A25904DG, MA, USA). Conditioned medium of BMSCs was gathered and centrifuged (2500 × *g* for 30 min, 8500 × g for 30 min) at 4 °C to remove cell debris, followed by centrifugation at 120,000 × *g* for 140 min (4 °C) using a Hitachi ultracentrifuge CS150FNX (Tokyo, Japan). The pellets were then washed by phosphate-buffered saline (PBS) under centrifugation of 120,000 × *g* (4 °C) for 70 min and stored at −80 °C.

### Characterization of exosomes

The particle size and concentration of BMSC-Exo were examined using ZetaView PMX 110 (Meerbusch, Germany). The morphology of BMSC-Exo was observed and photographed under a Hitachi TEM (Tokyo, Japan). Exosomal and cytoplasmic biomarkers were detected by western blot.

### Exosome uptake experiment

HA-VSMCs were seeded in a 12-well plate and 100 μL of PKH67 (Sigma-Aldrich, cat. PKH67GL, MI, USA) labeled exosomes were added and incubated for 30 min, 6 h, or 24 h. Then the microfilaments were marked with rhodamine-phalloidin (Cytoskeleton, Denver, CO, USA), and cells were stained with DAPI before observation by confocal microscope (Olympus, Tokyo, Japan).

### Transfection of mimics, inhibitors, siRNAs, and plasmids

Hsa-miR-381-3 mimics (miR10000736-1-5)/inhibitors (miR20000736-1-5) and mimics NC/inhibitors NC were obtained from RiboBio (Guangzhou, China) and transfected into HA-VSMCs/ BMSCs by riboFECTCP transfection kit (RIBO, Guangzhou, China). SiRNA targeting human NFAT5 (Si-NFAT5) and scrambled siRNA (Si-NC) were obtained from GenePharm (Shanghai, China). Human NFAT5 over-expression plasmid (oe-NFAT5, recombinant pcDNA3.1/NFAT5 plasmid) and pcDNA3.1-vector (ve) were obtained from Youbio (Hunan, China).

### Animals

The 5/6 SNx rat model of CKD was established in accordance with the method published by Ghosh et al. [42]. Twenty-four healthy Sprague Dawley rats (12-week-old male SD rats weighing 380 ± 20 g) were purchased from Beijing Wei Shang Li De Biological Technology Company (Beijing, China), and were randomly divided into 3 experimental groups referred to the studies from Ignacio Lopez et al. [43] and Aquiles Jara et al. [44, 1] sham-operation + normal diet (0.6% Ca–0.9% Pi) (group SHAM, *n* = 8); [2] 5/6 nephrectomy + high phosphate diet (HPD, 0.6% Ca–1.2% Pi) + calcitriol (80 ng/kg) every other day intraperitoneally (group SNx, *n* = 8); [3] 5/6 nephrectomy + HPD + calcitriol + tail vein injection of BMSC-Exo (75 μg) twice a week (group SNx + Exo, *n* = 8).

At the 16th week of the experiment, all animals received anesthesia followed by surgical exposure and removal of the thoracic aorta. Serum and thoracic aorta were immediately collected and kept at −80 °C.

### Patient information and sample collection

Patient artery samples. A 2-4-mm segment of the artery (radial artery, ulnar artery or, brachial artery) was excised from CKD patients who underwent an arterial venous fistula (AVF) operation (*n* = 20) for dialysis. Samples were then stored at −80 °C or in 4% paraformaldehyde before use.

### Alizarin Red S staining and Von Kossa (V-K) staining

HA-VSMCs and thoracic aorta sections were stained with 1% Alizarin Red S (Solarbio Science & Technology, cat. G1452, Beijing, China) for 15–30 min after fixation with 4% paraformaldehyde. For V-K staining, the sections of thoracic aorta were treated with 2% silver nitrate solution under ultraviolet radiation for 30 min.

### Ca^2+^ content and alkaline phosphatase (AKP) activity assay

Cells or tissues were disrupted by ultrasound and were detected by Ca^2+^ content and AKP activity assay according to the manufacturer’s instructions (Nanjing Jiancheng Bio. Institute, C004-2-1 and A059-2-2, Nanjing, China).

### TUNEL apoptosis assay

Apoptosis of the thoracic aorta was detected by Promega G3250 DeadEnd™ Fluorometric TUNEL System (WI, USA). Briefly, Proteinase K was used to permeabilize the thoracic aortas. The thoracic aortas were then incubated in the Terminal Deoxynucleotidyl Transferase (TdT) solution for 60 min and 2X SCC was used to stop the reaction. DAPI was used to visualize the nuclei.

### Flow cytometry assay

Cells were incubated with annexin-V FITC/PI (BD Biosciences, NJ, USA) for 15 min and analyzed with FACSCalibur Flow Cytometer (BD Biosciences, NJ, USA). Data were processed using FlowJo OSX 10.6 (BD Biosciences, NJ, USA).

### Western blot

Protein samples from BMSC-Exo, cells, and tissues were extracted, followed by quantification using PierceTM BCA Protein Assay kit (Thermo Fisher Scientific, MA, USA). The primary antibodies were listed in Supplementary Table 1.

### RT-qPCR

RNA samples were extracted using TRIzol reagent (Thermo Fisher, cat. 15596026, MA, USA), followed by reverse transcription using the Reverse Transcription System (Roche, Basel, Switzerland) or miRNA First-Strand cDNA Synthesis Kit (Tiangen, cat. KR211, Beijing, China). RT-qPCR was performed using SYBR Green Master Mix reagents (Life Technologies, CA, USA). Primers were listed in Supplementary Table 2.

### Microarray assay

A total RNA microarray assay was performed to identify differentially expressed genes (DEGs) and miRNAs (DEMs) in HA-VSMCs under different stimulations. RNA microarrays were performed by Oebiotech (Shanghai, China).

### Immunohistochemistry

Arteries sections were placed in citric saline for antigen repair. After blocking with 0.3% hydrogen peroxide and goat serum (ZSGB-Bio, ZLI-9022, Beijing, China), primary antibodies against NFAT5 (Abcam, ab3446, 1:100) were applied, followed by incubation with biotinylated secondary antibody.

### Dual-luciferase reporter gene assay

The sequences of wild (Wt) and mutant type (Mut) of NFAT5 3’untranslated region (UTR) were synthesized and cloned into the pMIR-REPORT vector (SyngenTech, Beijing, China). The vectors and miR-381-3p mimics were co-transfected into HEK-293T cells. The luciferase activity was measured with Dual- Luciferase^®^ Reporter Assay system (Promega, China).

### Statistical analysis

SPSS Version 20 (IBM Inc. Chicago, USA) and GraphPad Prism 6.0 were used for data analysis. Means and standard deviation (SD) were calculated for all data points, from at least three independent experiments. Significant differences between two groups were determined by independent-sample Student’s *t* test. One-way factorial ANOVA with Tukey’s post-hoc test was used for groups >2. *P* < 0.05 was considered significant.

## Supplementary information

Supplementary materials 1

Supplemental Material-2

Supplemental Material-3

cdd-author-contribution-form

aj-checklist

## Data Availability

The datasets used and analyzed during the current study are available from the corresponding author on reasonable request.
