# Supplementary material for: Bone marrow mesenchymal stem cell-derived exosomal microRNA-381-3p alleviates vascular calcification in chronic kidney disease by targeting NFAT5
Source: Cell Death Dis. 2022 Mar 28;13(3):278. doi: 10.1038/s41419-022-04703-1 (PMC8964813; doi:10.1038/s41419-022-04703-1)
Supplement: Supplementary file 1 — Supplementary materials 1 [file 41419_2022_4703_MOESM1_ESM.pdf]

Table S1. Primary antibodies used in Western Blot

Primary antibody	Manufacturer	Dilution
anti-Alix	ProteinTech, 12422-1-AP, Wuhan, China	1:2000
anti-TSG101	Abcam, ab125011, MA, USA	1:1000
anti-CD63	Abcam, ab134045, MA, USA	1:1000
anti-Calnexin	Abcam, ab133615, MA, USA	1:1000
anti-GM130	ProteinTech, 11308-1-AP, Wuhan, China	1:2000
anti-GAPDH	ProteinTech, 60004-1-Ig, Wuhan, China	1:5000
anti-NFAT5	Santa Cruz, sc-398171, CA, USA	1:1000
anti-BAX	Abcam, ab32503, MA, USA	1:1000
anti-BCL 2	Abcam, ab32124, MA, USA	1:1000
anti-Caspase 3	ProteinTech, 19677-1-AP, Wuhan, China	1:1000

Table S2. Primers for RT-qPCR

Gene		Sequence (5’-3’) or product number
Human- BAX	Forward	GGAGGAAGTCCAATGTCCAG
	Reverse	GGGTTGTCGCCCTTTTCTAC
Human- Bcl 2	Forward	GAGAAATCAAACAGAGGCCG
	Reverse	CTGAGTACCTGAACCGGCA
Human- NFAT5	Forward	GGGTCAAACGACGAGATTGTG
	Reverse	GTCCGTGGTAAGCTGAGAAAG
Human- β-action	Forward	TGACGTGGACATCCGCAAAG
	Reverse	CTGGAAGGTGGACAGCGAGG
Rat- BAX	Forward	GGCGATGAACTGGACAACAA
	Reverse	GCAAAGTAGAAAAGGGCAACC
Rat- Bcl 2	Forward	ACAGAGGGGCTACGAGTGGG
	Reverse	AGCGGGCGTTCGGTTG
Rat- NFAT5	Forward	CAGATCCAGCTGGTGCTTTGAG
	Reverse	TATGCCTTGGGCTGAAGCTG
Rat- β-action	Forward	GAAGTGTGACGTTGACATCCG
	Reverse	GCCTAGAAGCATTTGCGGTG
miR-381-3p	Forward	Ribobio: miRA1000269-1-100
miR-17-5p	Forward	Ribobio: miRA0000070-1-100
miR-210-3p	Forward	Ribobio: miRA0000267-1-100
miR-20a-5p	Forward	Ribobio: miRA0000075-1-100
U6	Forward	Ribobio: miRAN0002-1-100

Figure S1. (A-C) Quantitative results of Alizarin Red staining. (D-E) The mRNA and protein expression levels of NFAT5 were detected after transfection of si-NFAT5. (F) Expression of miR-381-3p was detected by RT-qPCR. (G-H) Alizarin Red S staining, Ca^{2+} content, and AKP activity results were detected after transfection of si-NFAT5 (scale bar = 100μm). (I) Apoptosis ratio was measured by flow cytometry. (J-L) Western blot and qRT-PCR was used to detect the mRNA and protein expression of BAX, BCL-2 and cleaved caspase-3. *$P < 0.05$ compared with the Si-NC group; #$P < 0.05$ compared with the Pi+Si-NC group.

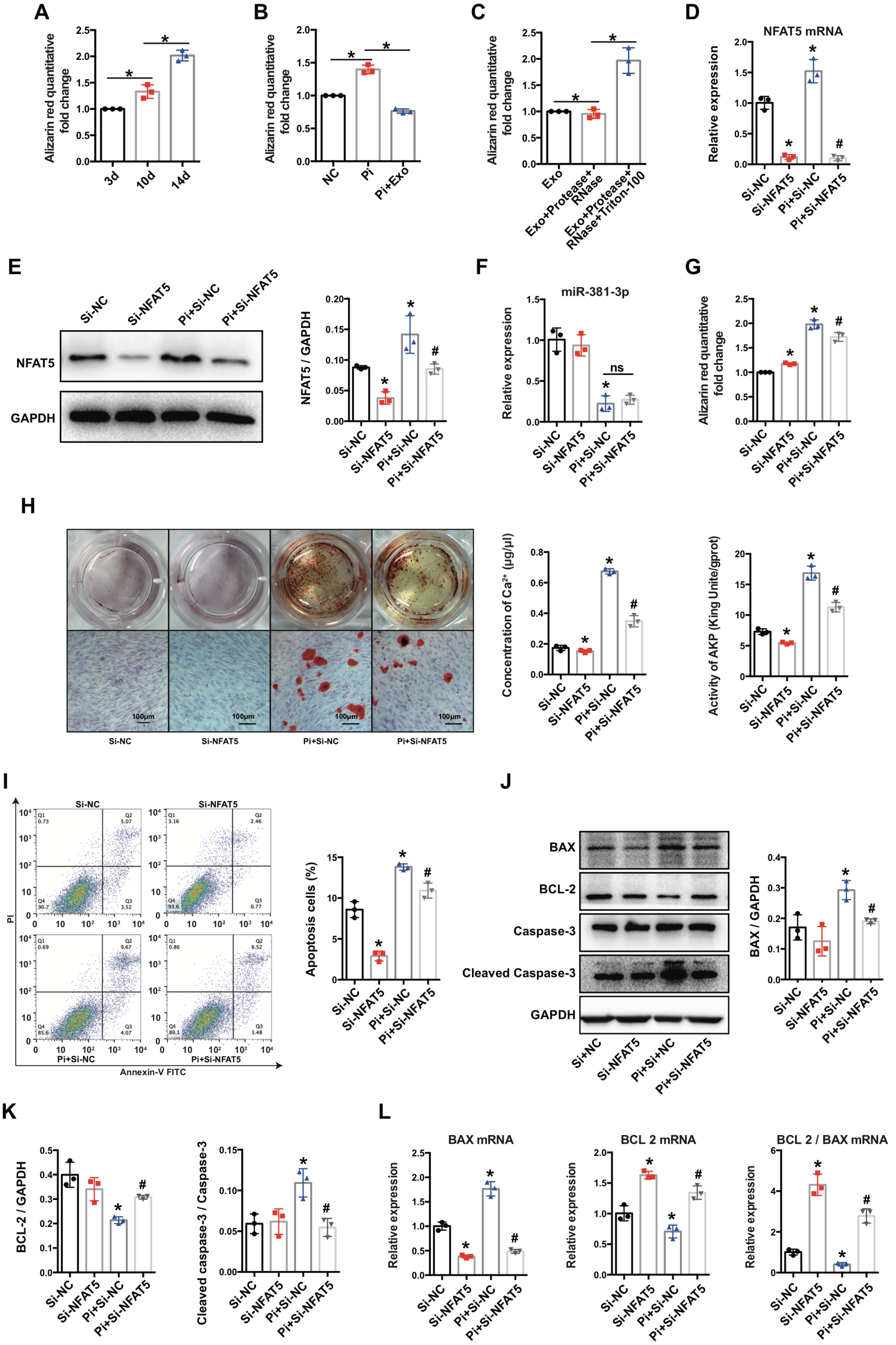

A
Alizarin red quantitative fold change
3d
10d
14d
B
NC
Pi
Pi+Exo
C
Exo
Exo+Protease+ RNase
Exo+Protease+ RNase+Triton-100
D
NFAT5 mRNA
Relative expression
Si-NC
Si-NFAT5
Pi+Si-NC
Pi+Si-NFAT5
E
NFAT5
GAPDH
NFAT5 / GAPDH
F
miR-381-3p
ns
G
H
100μm
Concentration of Ca2+ (μg/μl)
Activity of AKP (King Unite/gprot)
I
PI
Annexin-V FITC
Apoptosis cells (%)
J
BAX
BCL-2
Caspase-3
Cleaved Caspase-3
BAX / GAPDH
K
BCL-2 / GAPDH
Cleaved caspase-3 / Caspase-3
L
BAX mRNA
BCL 2 mRNA
BCL 2 / BAX mRNA
