# Supplementary material for: Bone marrow mesenchymal stem cell-derived exosomal microRNA-381-3p alleviates vascular calcification in chronic kidney disease by targeting NFAT5
Source: Cell Death Dis. 2022 Mar 28;13(3):278. doi: 10.1038/s41419-022-04703-1 (PMC8964813; doi:10.1038/s41419-022-04703-1)

Figure 1C

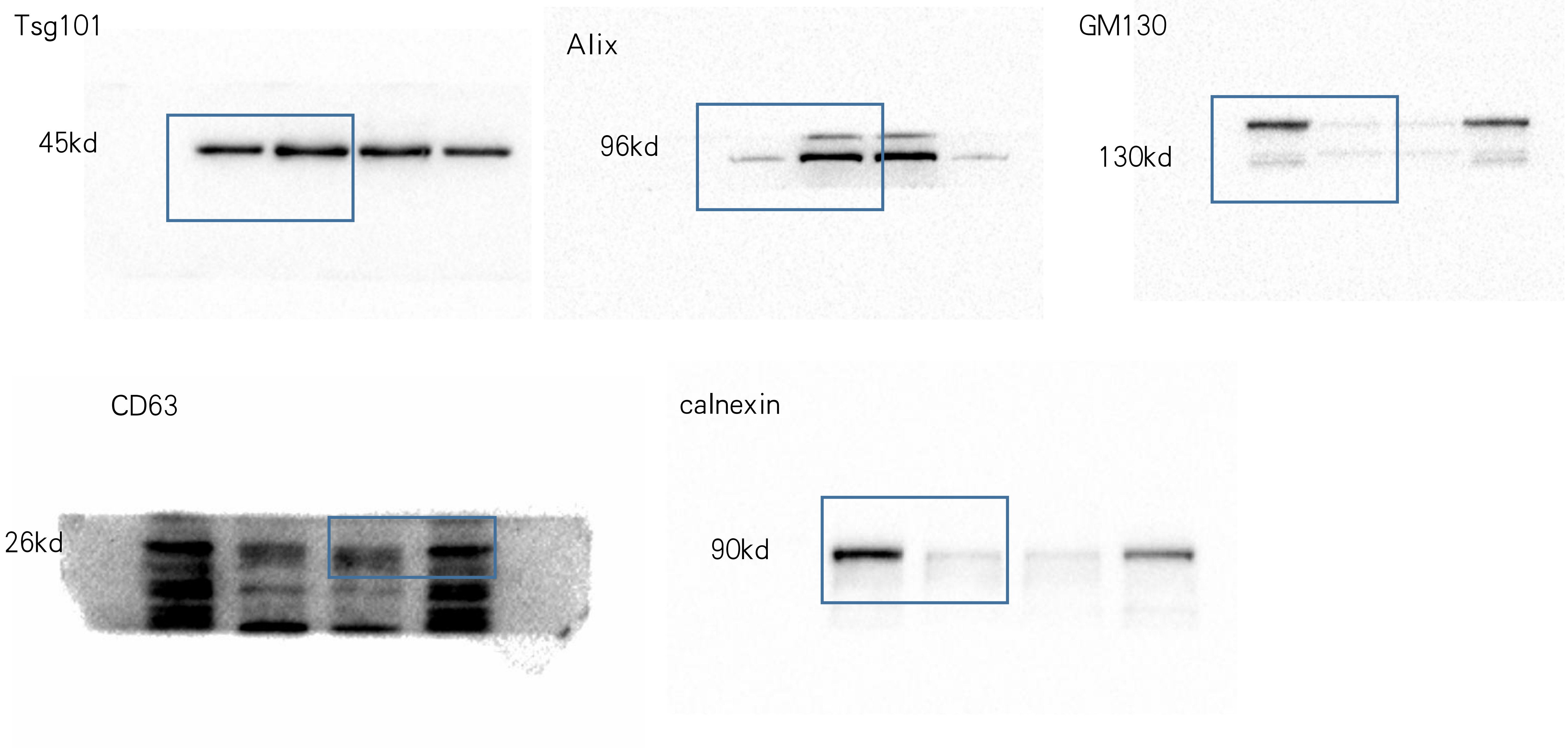

Figure 1G

bax
21kd

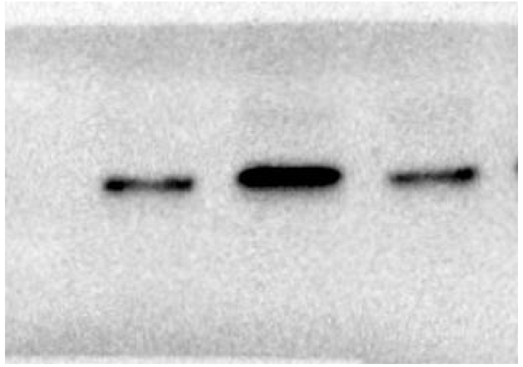

bcl 2
26kd

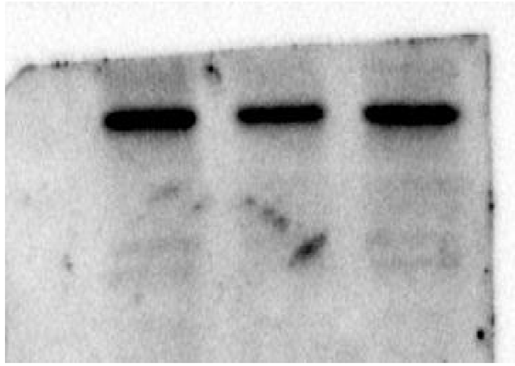

gapdh
36kd

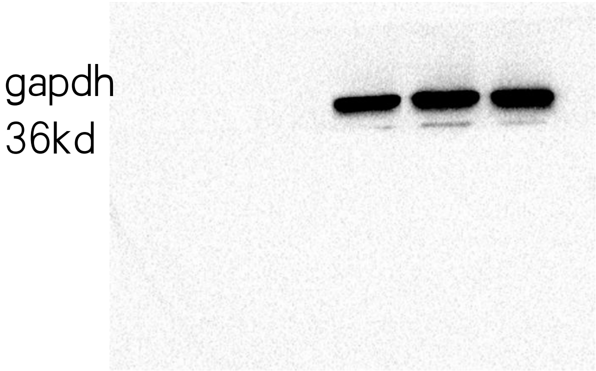

cas3
32kd

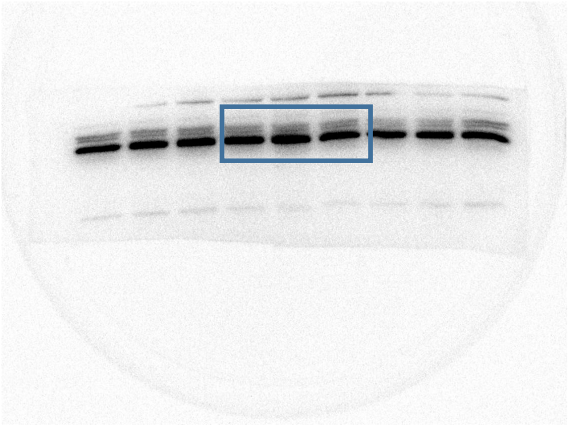

cleaved cas3
17kd

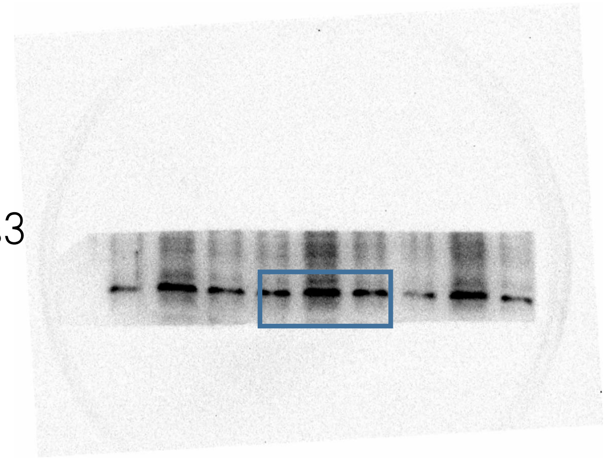

Figure 2G

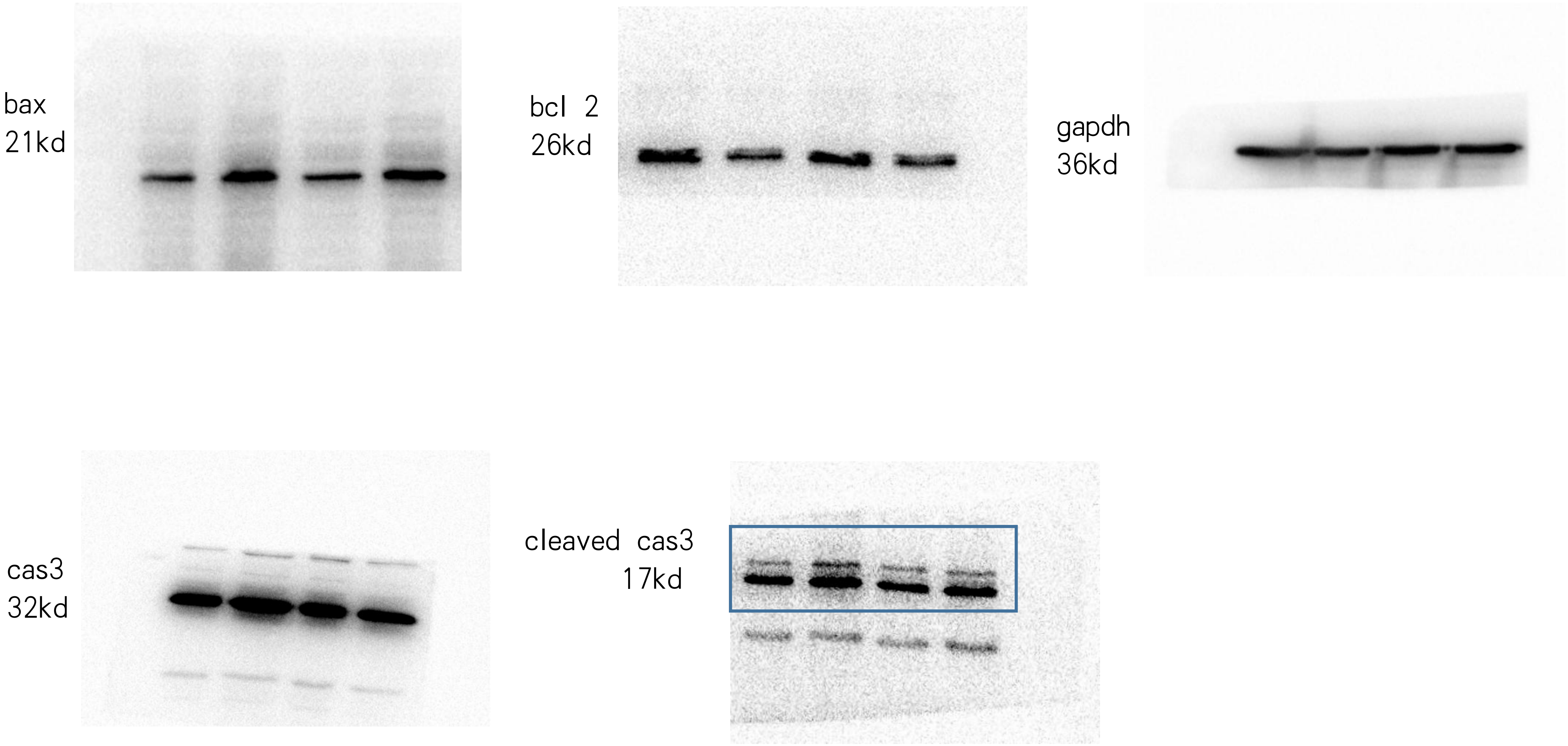

Figure 3G

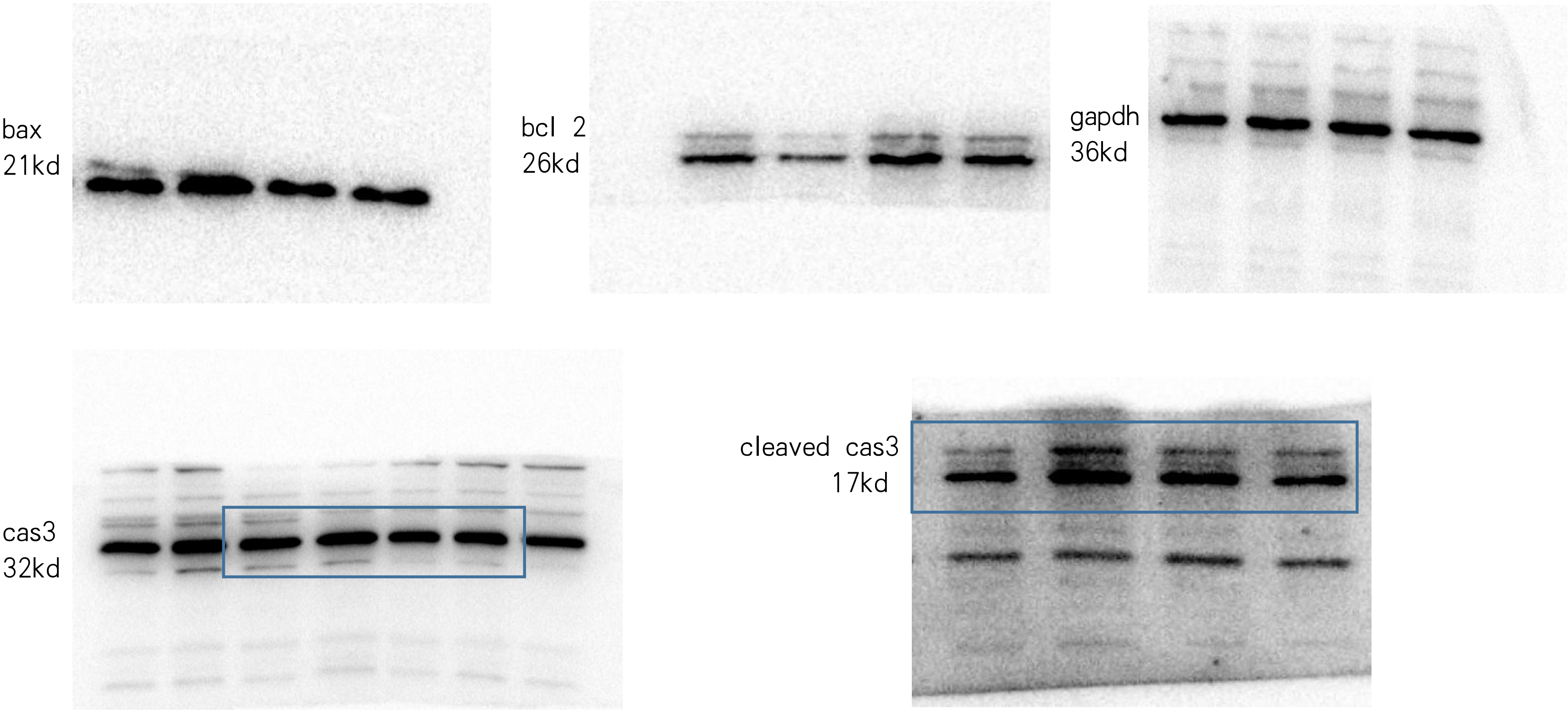

Figure 4F

NFAT5
150kd

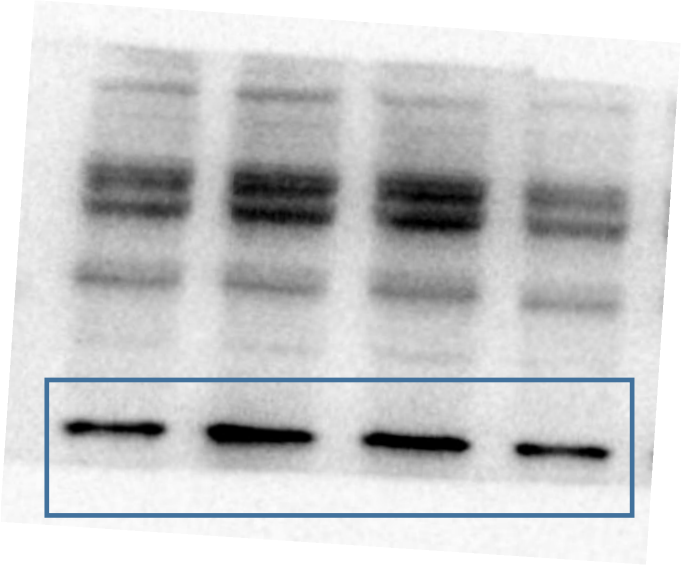

gapdh
36kd

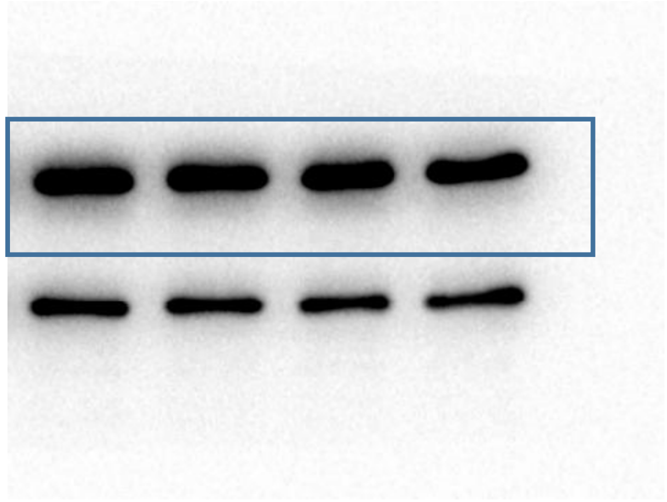

Figure 4H

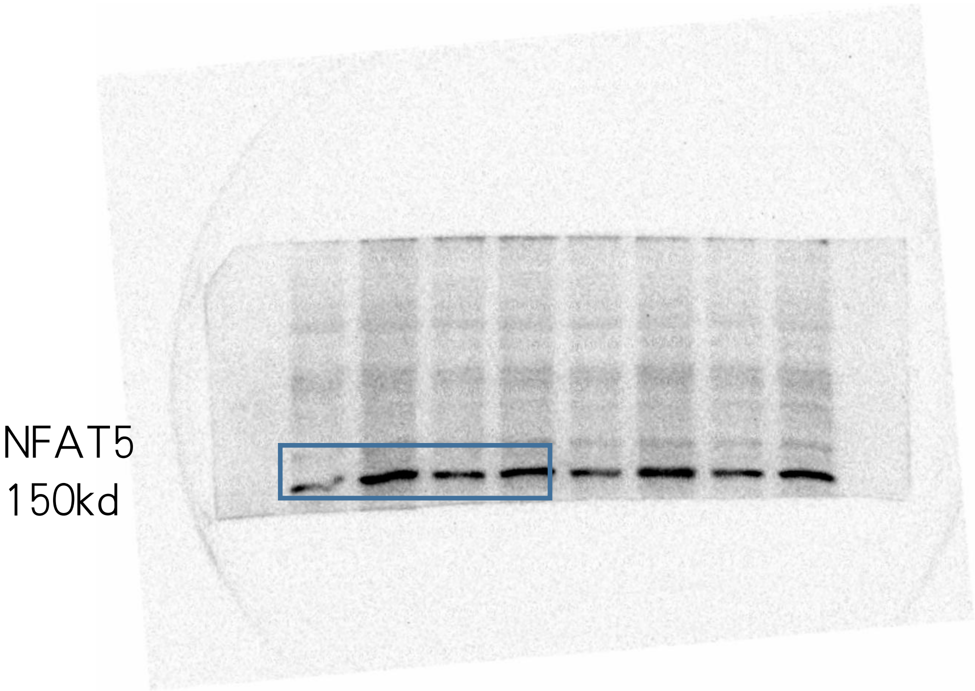

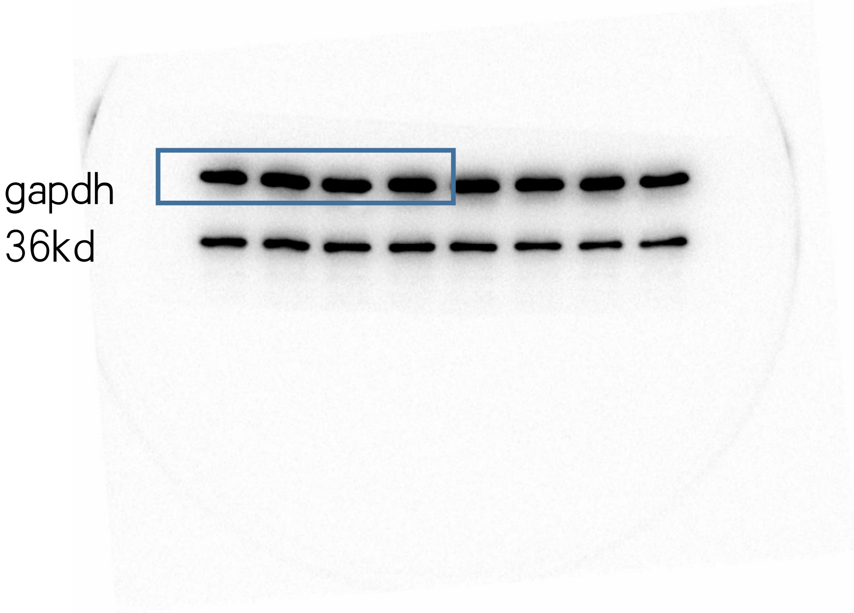

Figure 5B

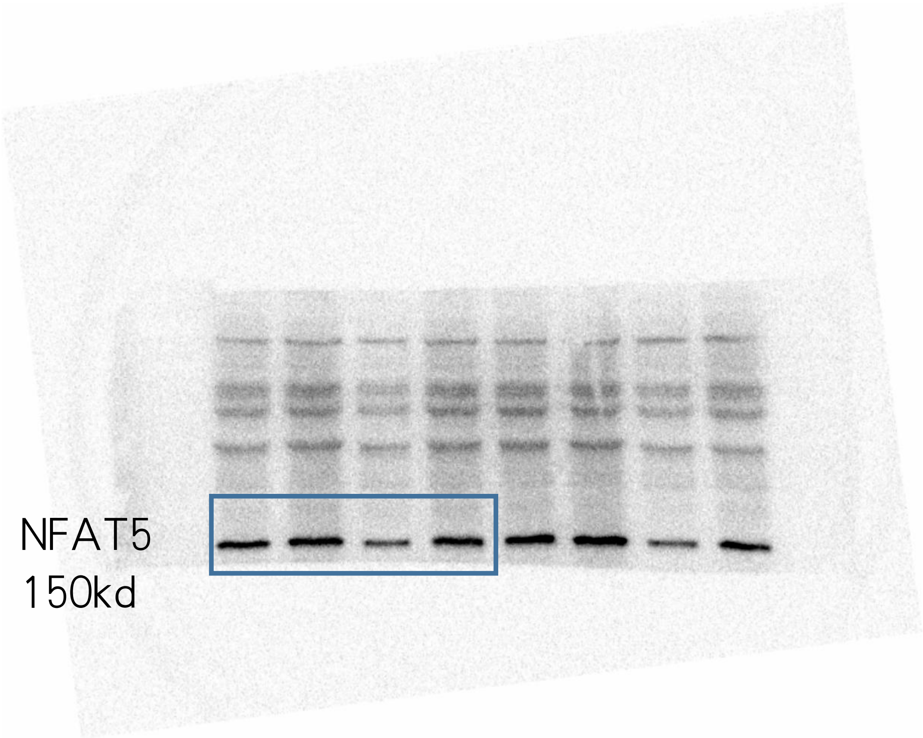

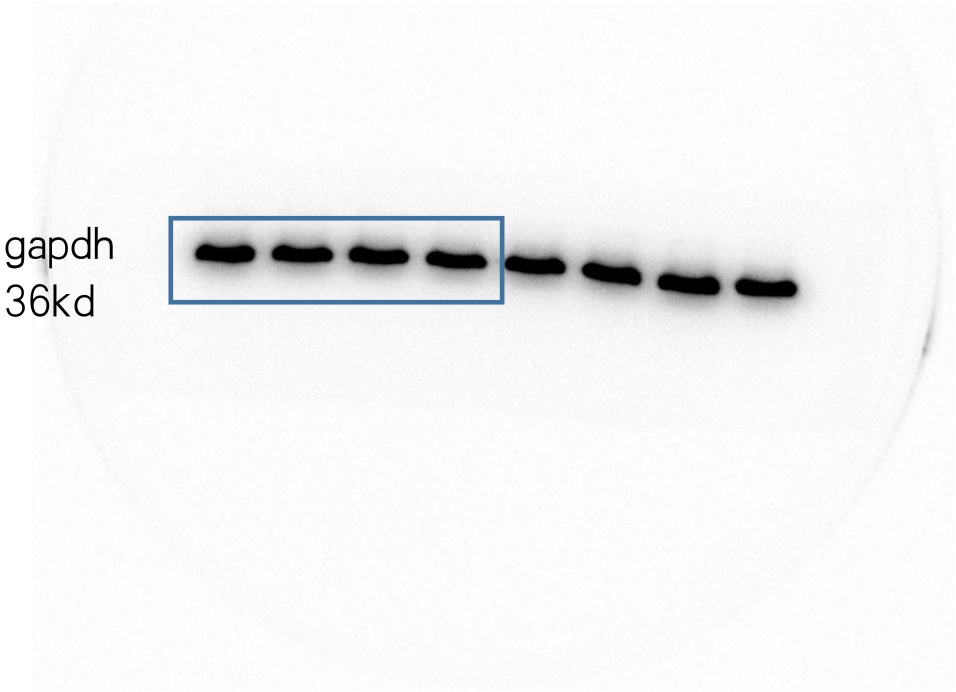

Figure 5H

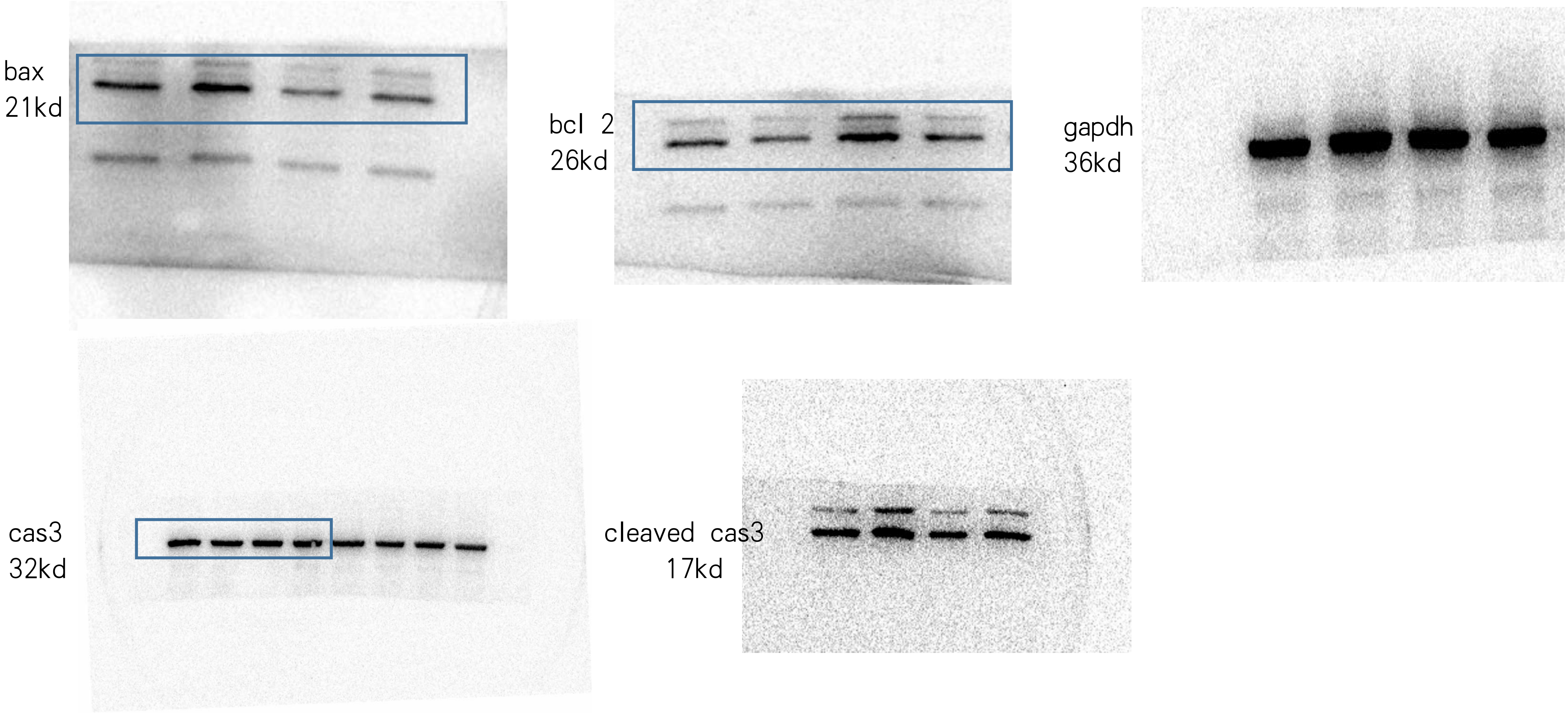

Figure 7B

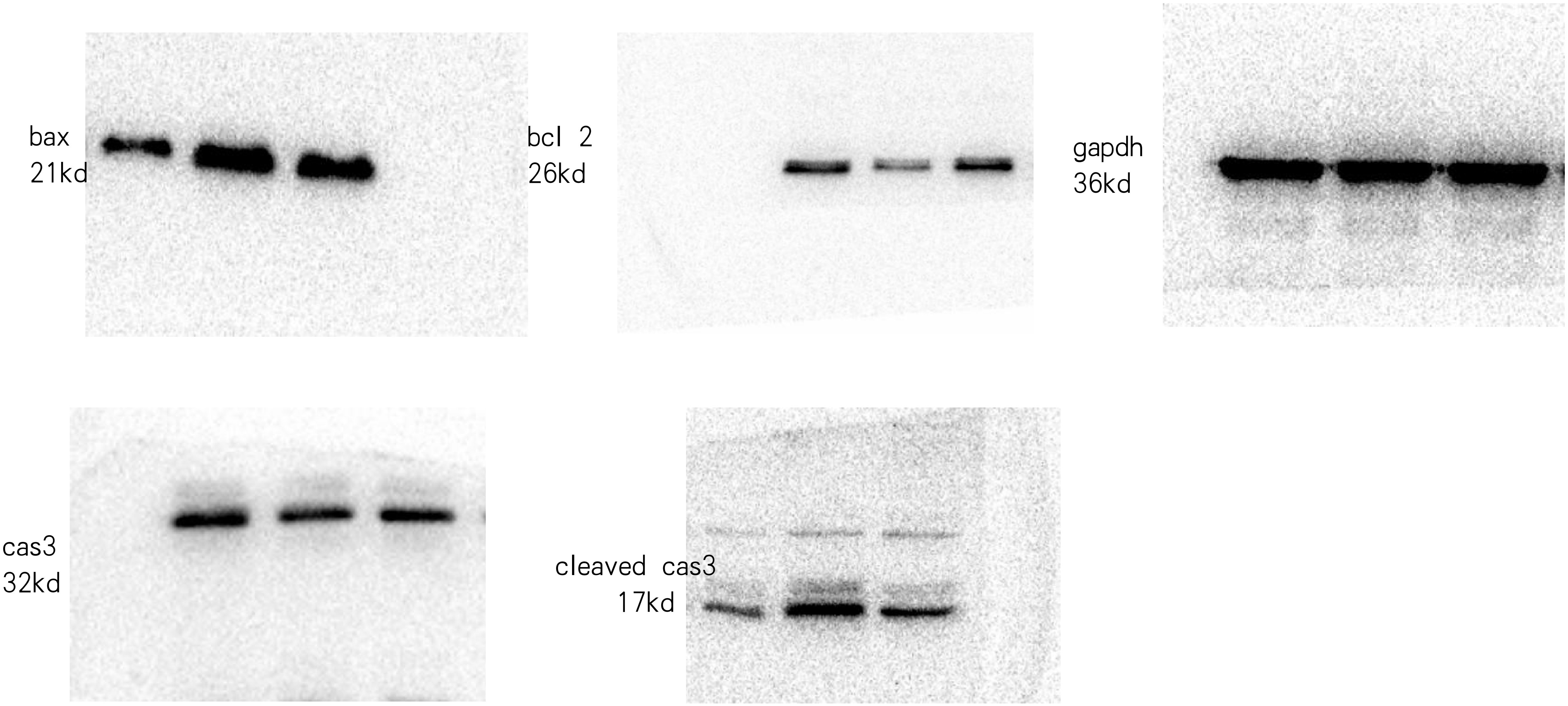

Figure 7E

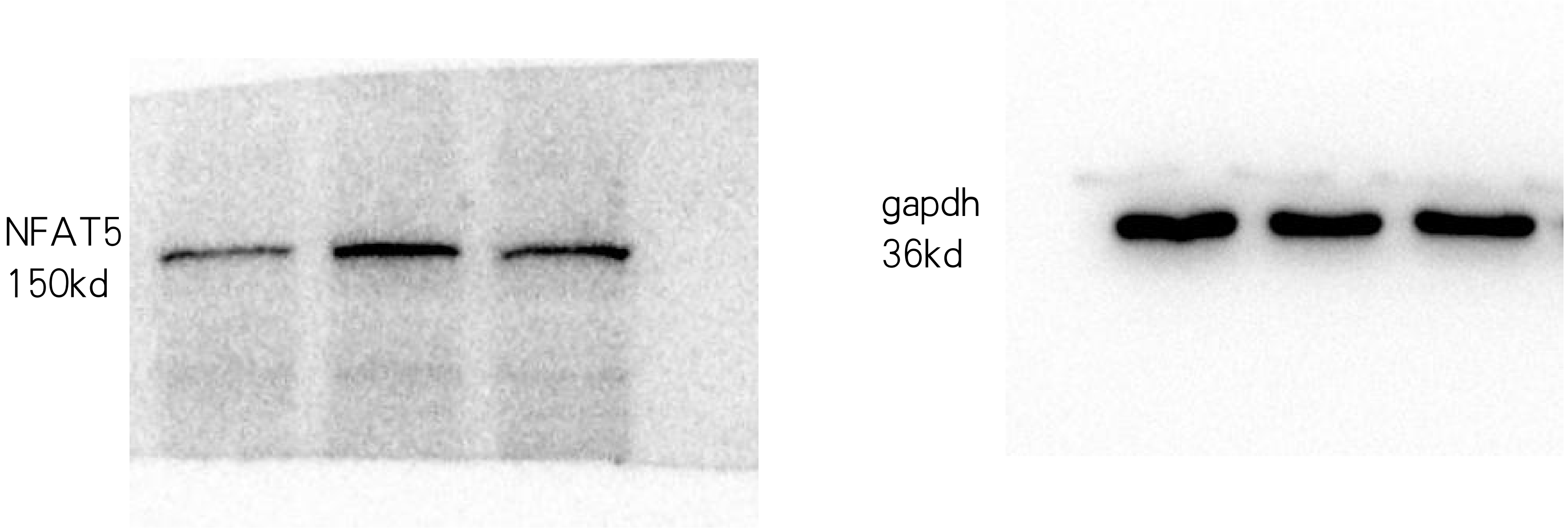

Supplement: Supplementary file 2 — Supplemental Material-2 [file 41419_2022_4703_MOESM2_ESM.pdf]
