# Supplementary material for: Bone marrow mesenchymal stem cell-derived exosomal microRNA-381-3p alleviates vascular calcification in chronic kidney disease by targeting NFAT5
Source: Cell Death Dis. 2022 Mar 28;13(3):278. doi: 10.1038/s41419-022-04703-1 (PMC8964813; doi:10.1038/s41419-022-04703-1)
Supplement: Supplementary file 3 — Supplemental Material-3 [file 41419_2022_4703_MOESM3_ESM.pdf]

edanz 理文编辑
INNOVATIVE SCIENTIFIC SOLUTIONS

Certificate of Editing

Edited provisional title

Bone mesenchymal stem cell-derived exosomal microRNA-381-3p alleviates vascular calcification in chronic kidney disease by targeting NFAT5

Client name and institution

Yingjie Liu, Department of Nephrology, Faculty of Kidney Diseases, Beijing Friendship Hospital, Capital Medical University, Beijing, China

Date Completed	Identification code
2021-07-07	98040

Certificate issued by
Koji Yamashita
Managing Director and CEO

Expert Editor: Emma Longworth-Mills
2019 PhD Anatomy & Cell Biology
Indiana University School of Medicine
Biochemistry and Cell Biology, Medical Physiology, Neurosciences

www.liwenbianji.cn

While this certificate confirms the authors have used Edanz's editing services, we cannot guarantee that additional changes have not been made after our edits.
