# Supplementary material for: Bone marrow mesenchymal stem cell-derived exosomal microRNA-381-3p alleviates vascular calcification in chronic kidney disease by targeting NFAT5
Source: Cell Death Dis. 2022 Mar 28;13(3):278. doi: 10.1038/s41419-022-04703-1 (PMC8964813; doi:10.1038/s41419-022-04703-1)
Supplement: Supplementary file 4 — cdd-author-contribution-form [file 41419_2022_4703_MOESM4_ESM.pdf]

DECLARATION OF CONTRIBUTIONS TO ARTICLE

ADMC

Manuscript Number: CDDIS-21-3233-T

Journal Name: Cell Death & Disease (the 'Journal')

Proposed Title of the Contribution: (the 'Contribution)

Author(s): Yingjie Liu, Yan Guo, Shumin Bao, Hongdong Huang, Wenhu Liu, Weikang Guo (the 'Authors')

For all *CDD* articles, each person named as an author in the published version must be able to show he or she has contributed substantially to the article.

Authorship credit should be based on 1) substantial contributions to conception and design, acquisition of data, or analysis and interpretation of data; 2) drafting the article or revising it critically for important intellectual content; and 3) final approval of the version to be published. Authors should meet conditions 1, 2 and 3.

Any person who cannot be shown to have made a substantial contribution to the article cannot be listed as an author in the final version. The name of any person who is deemed to have made a minor contribution can, however, appear in the Acknowledgments section of the article.

Please complete the table below to indicate the contributions of all named authors to the manuscript.

Author Full Name:	Specification of Contribution to the Manuscript:
Yingjie Liu	study concept and design, wrote the manuscript
Yan Guo	interpretation of data and statistical analysis
Shumin Bao	interpretation of data and statistical analysis
Hongdong Huang	study concept and design
Wenhu Liu	study concept and design
Weikang Guo	study concept and design

Please complete the table below to indicate the contributions of all named authors to the figures.

Figure 1:
Yingjie Liu generated the data. YaWeikang Guo generated the immune-histochemistry data and labelled the image. Yingjie Liu assembled the figure.

Figure 2:
Yingjie Liu generated the data. Weikang Guo generated the immune-histochemistry data and labelled the image. Yingjie Liu assembled the figure.

Figure 3:
Yingjie Liu generated the data. Weikang Guo generated the immune-histochemistry data and labelled the image. Yingjie Liu assembled the figure.

Figure 4:
Yingjie Liu generated the data. Weikang Guo generated the immune-histochemistry data and labelled the image. Yingjie Liu assembled the figure.

Figure 5:
Yingjie Liu generated the data. Weikang Guo generated the immune-histochemistry data and labelled the image. Yingjie Liu assembled the figure.

Figure 6:
Yingjie Liu generated the data. Weikang Guo generated the immune-histochemistry data and labelled the image. Yingjie Liu assembled the figure.

Signed for and on behalf of the Author(s):	Print Name:	Date:
Wenhu Liu	Wenhu Liu	2021/08/11
